# Psychological and functional predictors of chronic pain outcomes in youth with Duchenne muscular dystrophy

**DOI:** 10.3389/fpubh.2026.1758612

**Published:** 2026-01-14

**Authors:** Ruihao Li, Li Gao, Turong Chen, Chunming Zhou, Meihuan Huang

**Affiliations:** Department of Rehabilitation Medicine, Shenzhen Children’s Hospital, Shenzhen, China

**Keywords:** chronic pain, Duchenne muscular dystrophy, longitudinal predictors, pain outcomes, pediatrics

## Abstract

**Objectives:**

This study aimed to characterize 1-year pain trajectories in children with Duchenne muscular dystrophy (DMD) and identify predictors across physical, psychosocial, and quality-of-life domains.

**Methods:**

We conducted a 1-year prospective cohort study involving young males with genetically confirmed DMD. Baseline assessments included sociodemographic characteristics, clinical history, chronic pain profile, fatigue, psychological distress, and health-related quality of life (HRQoL). Follow-up assessments were completed approximately 1 year later through structured telephone interviews with participants and their primary caregivers. Outcomes at follow-up included the presence of chronic pain, pain intensity, and pain interference.

**Results:**

A total of 73 participants (median age = 10.6 years; range = 8–16 years) completed the 1-year follow-up assessment. Multivariable logistic regression analysis indicated that physical function (*p* = 0.028; OR = 0.929, 95% CI [0.586, 0.988]) and psychological distress (OR = 1.502, 95% CI [1.157, 5.508], *p* = 0.004) were significant independent predictors of chronic pain. Physical function also demonstrated a modest indirect effect through psychological distress (indirect effect = −0.048, 95% CI [−0.369, −0.0004]). Fatigue (indirect effect = −0.116, 95% CI [−0.567, −0.038]) and HRQoL (indirect effect = −0.064, 95% CI [−0.428, −0.022]) influenced pain outcomes only indirectly via psychological distress. Multivariable linear regression further showed that both psychological distress (severity: *B* = 0.208, *p* < 0.001; 95% CI [0.105, 0.307]; interference: *B* = 0.193, *p* < 0.001; 95% CI [0.100, 0.279]) and fatigue (severity: *B* = −0.054, *p* = 0.037; 95% CI [−0.107, −0.006]; interference: *B* = −0.047, *p* = 0.020; 95% CI [−0.085, −0.008]) were associated with increased pain burden. Physical function predicted pain severity (*B* = −0.036, *p* = 0.001; 95% CI [−0.057, −0.012]) but not pain interference, whereas HRQoL did not show a significant effect in either model.

**Conclusion:**

This prospective study identifies psychological distress and reduced physical function as independent contributors to chronic pain outcomes in DMD, with psychological distress mediating the effects of fatigue and HRQoL. Physical function was primarily associated with pain severity, whereas psychological distress and fatigue predicted the broader pain burden, including functional interference. These findings support a biopsychosocial model in which psychosocial factors translate underlying biological vulnerability into clinically significant pain.

## 1 Introduction

Duchenne muscular dystrophy (DMD) is a severe X-linked neuromuscular disorder characterized by progressive muscle degeneration, loss of ambulation, and multi-systemic complications (1). Beyond the hallmark motor impairments, chronic pain has emerged as a prevalent yet under-recognized symptom that significantly compromises patients’ quality of life (2–4). Our previous systematic review and meta-analysis showed that up to 60% of individuals with muscular dystrophies experience chronic pain, and 62% (95% CI: 50–73%) of patients with DMD, across both pediatric and adult populations, report pain in various anatomical regions, including the back, lower limbs, and joints (3). These findings were further corroborated in our later prevalence study of Chinese children with DMD, in which 40% of participants reported persistent or recurrent chronic pain, underscoring the universality of this issue across different cultural and healthcare contexts (5).

Despite its high prevalence, pain in DMD remains inadequately assessed and poorly managed in routine clinical care (5–7). This may reflect a persistent view that pain is an inevitable and secondary consequence of disease progression. Clinically, pain has often been attributed primarily to mechanical factors such as muscle contractures, postural deformities, or immobilization, rather than being acknowledged as a complex, multidimensional symptom requiring systematic assessment and targeted management (7, 8). However, emerging evidence indicates a far more complex and multidimensional etiology (5, 9). Recent studies found that children with DMD who report chronic pain exhibit significantly higher psychological distress, increased fatigue, and worse health-related quality of life (HRQoL), suggesting that emotional and cognitive domains may interact with physical impairments to shape the pain experience (5, 9). Furthermore, our study on somatosensory profiling using quantitative sensory testing (QST) demonstrated widespread alterations in mechanical pain thresholds in patients with DMD, including in individuals without clinical pain complaints, suggesting underlying heightened pain sensitization that may predispose certain individuals to chronic pain (9).

Previous studies have identified cross-sectional associations between chronic pain and various physical or emotional factors in individuals with DMD (5, 9); however, the causal mechanisms remain unclear, and longitudinal evidence that may predict pain outcomes is limited. The trajectories of chronic pain over time in DMD are still largely unexplored, with an observed absence of prospective cohort studies investigating predictors of long-term pain experience. Despite growing acknowledgment of the multidimensional nature of pain, no study to date has systematically evaluated the collective role of physical, psychological, and psychosocial factors in shaping different pain pathways in this population. Identifying such predictors is crucial for risk stratification and for designing targeted, multidomain interventions.

In this study, we aimed to characterize 1-year pain trajectories in children with DMD and to identify key predictors from multiple domains, including physical function, fatigue, emotional wellbeing, and HRQoL. By integrating these physical and psychosocial variables within a comprehensive analytical framework, this study seeks to advance the understanding of pain progression in DMD and provide an evidence base for individualized and proactive pain management strategies.

## 2 Materials and methods

### 2.1 Participants and study design

This prospective cohort study involved young males aged 8–18 years with genetically confirmed DMD. Participants were recruited via consecutive sampling from the pediatric rehabilitation clinic at Shenzhen Children’s Hospital between October 2022 and December 2024. Patients who had cognitive impairments or speech and language dysfunction, as determined by the treating physician’s clinical judgment, that hindered their comprehension of pain questions or self-expression of pain complaints were excluded. This study (registry number: ChiCTR2300072100) was approved by the Ethics Committee for Medical Research of Shenzhen Children’s Hospital (202209602). Written informed consent was obtained from all parents or legal guardians, and written assent was obtained from participants capable of providing it, prior to participation.

### 2.2 Follow-up procedures and outcome measures

Participants were prospectively followed for 1 year. Baseline evaluations included sociodemographic data, clinical history, chronic pain profile, fatigue, psychological distress, and HRQoL. Questionnaires were primarily self-administered. However, for younger participants or those with significant upper limb limitations, parents were permitted to assist by reading questions aloud and recording the child’s responses. Clinical data collected, where available, encompassed: (1) age at symptom onset and diagnosis, genetic testing results, medication usage, and other treatments; (2) physical function, assessed via functional level, the standardized Motor Function Measure-32 Chinese version (MFM-32) (10), and documentation of wheelchair and orthotic use. Validated Chinese-language instruments previously utilized in this population were employed (5, 9): fatigue was assessed by the PedsQL™ Multidimensional Fatigue Scale (PedsQL™ MFS) (11); psychological distress by the Hospital Anxiety and Depression Scale (HADS) (12); and HRQoL by the Pediatric Quality of Life Inventory™ Version 4.0 Generic Core Scales (PedsQL™ 4.0) (13).

Pain presence and chronicity were evaluated using a structured questionnaire. Participants were initially asked, “Have you experienced any pain in the past 4 weeks, excluding occasional pain such as headache or toothache?” A positive response prompted a follow-up question regarding chronicity (14): “Did the pain start more than 3 months ago?” Those reporting pain were further queried about pain characteristics and indicated pain locations on a body map. Pain intensity over the previous week was measured using the Numeric Pain Rating Scale (NPRS), ranging from 0 (“no pain”) to 10 (“worst imaginable pain”) (15). To assess functional impact, a modified Pain Interference Scale from the Brief Pain Inventory (PIS-BPI) was administered, with adaptations to improve relevance for pediatric and disabled populations by replacing “walking ability” with “mobility: ability to get around” and “normal work” with “school work” for participants aged 8–18 years (16).

Follow-up evaluations were conducted approximately 1 year after baseline (±2 weeks), via structured telephone interviews with both participants and their primary caregivers. Follow-up outcomes included presence of chronic pain, pain intensity, and pain interference.

### 2.3 Sample size calculation

An *a priori* power analysis was conducted to determine the sample size for the primary outcome of chronic pain at 1-year follow-up, using G*Power (Version 3.1) for a multivariable logistic regression. The calculation was designed to provide 80% power at a two-sided significance level of *α* = 0.05. The hypothesized odds ratio (OR) for the key predictor, psychological distress, was set at 2.0. This effect size was selected based on prior literature indicating that the OR for psychological predictors of chronic pain typically ranges from 1.5 to 2.5 (17, 18); a moderate-to-strong effect was deemed appropriate for the DMD population, who may experience heightened distress. Additional assumptions included an event rate of 40% (5) and a squared multiple correlation (*R*^2^) of 0.10 to account for covariates. The analysis indicated a minimum of 93 participants for the final analysis. To accommodate an estimated 15% attrition rate, the final recruitment target was set at 110 participants.

### 2.4 Statistical analyses

All statistical analyses were performed using IBM SPSS Statistics version 31.0 (IBM Corp., Armonk, NY, USA). Descriptive statistics were used to summarize demographic and clinical characteristics. Continuous variables were tested for normality using the Shapiro–Wilk test. Normally distributed variables were presented as means and standard deviations (SD), while non-normally distributed variables were reported as medians and interquartile ranges (IQR). Categorical variables were summarized as frequencies and percentages.

Univariate analyses (group comparisons between chronic pain vs. no chronic pain) were conducted to identify potential baseline predictors for pain outcomes. Variables selected for multivariable regression were informed by both univariate results (*p* < 0.20) and clinical relevance. In cases of high collinearity, such as between age and motor function, the most theoretically relevant variable was retained to minimize multicollinearity. Multivariable logistic regression was performed to identify independent baseline predictors of chronic pain presence at 1-year follow-up, adjusting for baseline pain status to account for initial pain variability. ORs and 95% confidence intervals (CIs) were reported, and model fit was evaluated using the Hosmer–Lemeshow goodness-of-fit test and pseudo-*R*^2^ statistics (Cox & Snell, Nagelkerke). Where appropriate, mediation analyses were conducted using Hayes’ PROCESS macro (version 5.0). Model 4 was applied to test indirect effects through psychological distress, controlling for relevant covariates.

To explore predictors of pain burden (pain severity and interference) at 1-year follow-up, Spearman correlation analyses were first conducted. Subsequently, multivariable linear regression was used to identify independent predictors. Given the limited sample size (*n* = 32), only four baseline variables were entered simultaneously into each model. Model assumptions were checked, including multicollinearity (via tolerance and variance inflation factor, VIF) and autocorrelation (Durbin–Watson statistic). A two-tailed *p*-value < 0.05 was considered statistically significant for all analyses.

## 3 Results

### 3.1 Patient characteristics

**Figure 1:**
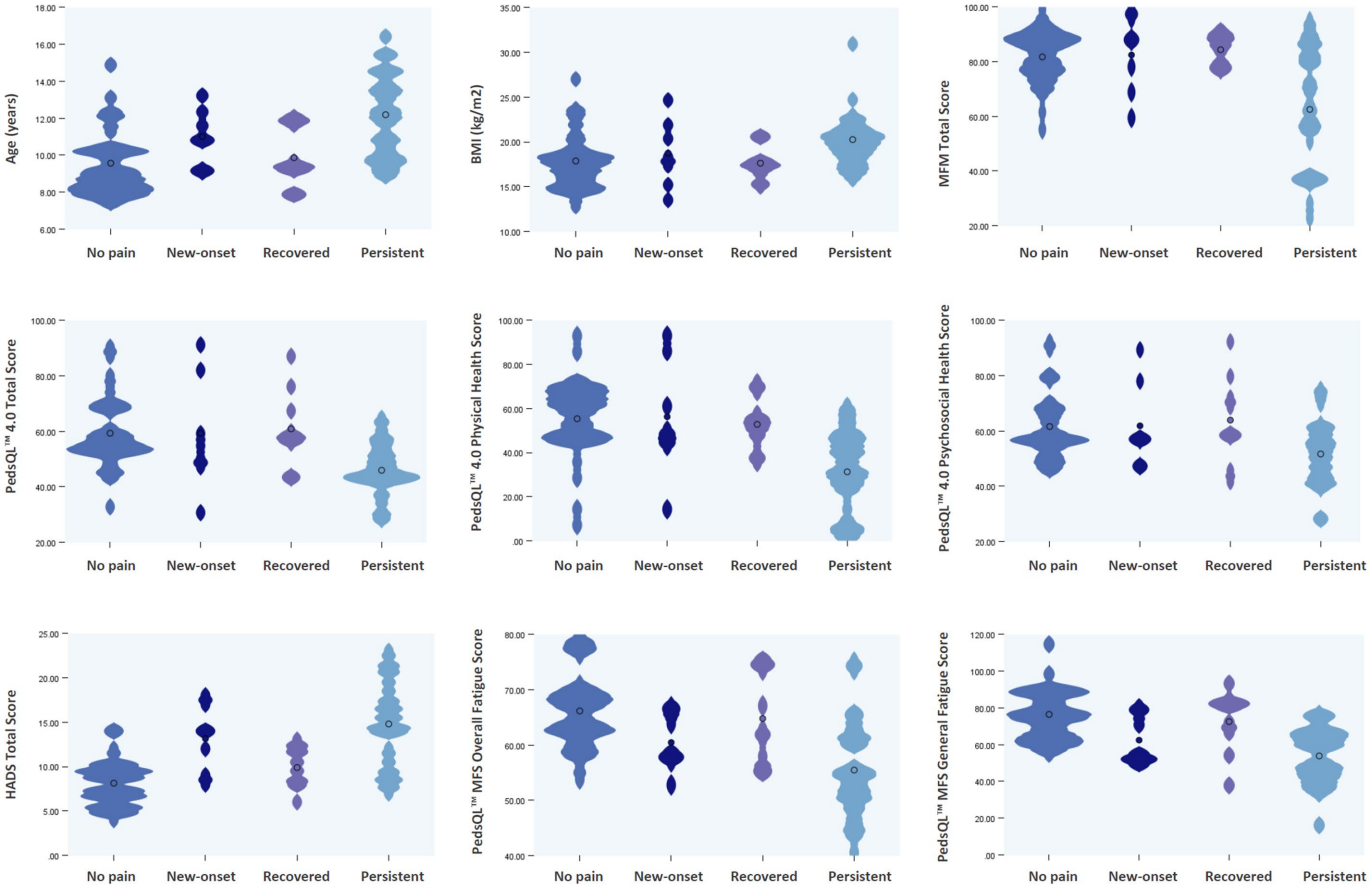
Violin plots illustrating the distribution of selected clinical variables across four pain trajectory groups. BMI, body mass index; MFM, Motor Function Measure; PedsQL™, Pediatric Quality of Life Inventory™ Scale; PedsQL™ MFS, Pediatric Quality of Life Inventory™ Multidimensional Fatigue Scale; HADS, Hospital Anxiety and Depression Scale.

From an initial cohort of 112 children with DMD, 21 were excluded at baseline due to cognitive impairments precluding pain self-report. Of the remaining 91 eligible participants, 73 completed the 1-year follow-up evaluation, yielding a retention rate of 80.2%. The final analytic sample had a median age of 10.6 years (range, 8–16). Participants were classified into four trajectory groups based on longitudinal changes in their pain profiles: 32 children (43.8%) remained pain-free throughout the study period (no pain), seven (9.6%) developed new chronic pain (new-onset chronic pain), nine (12.3%) recovered from baseline pain (recovered pain), and 25 (34.2%) experienced persistent pain at both time points (persistent chronic pain). For further analysis, these trajectories were dichotomized into two outcome groups: no chronic pain (combining the no pain and recovered pain groups; *n* = 41, 56.2%) and chronic pain (combining the new-onset and persistent chronic pain groups; *n* = 32, 43.8%). Baseline characteristics across pain outcome groups are presented in Table 1, with group-level distributions by pain trajectories illustrated using violin plots in Figure 1. Comparative analyses revealed significant differences in age, BMI, physical function, fatigue, psychological distress, and HRQoL between groups with and without chronic pain. Group differences were also found in the use of wheelchairs and orthotic devices, and in the severity of scoliosis.

**Table 1:** Baseline characteristics by pain outcome group.

Variables	Total	Pain outcome groups		*p*-value*
		No chronic pain	Chronic pain	
*N* (%)	73 (100%)	41 (56.2%)	32 (43.8%)	
Demographics				
Age, years, median (IQR)	10.2 (9.0, 12.2)	9.3 (8.2, 10.4)	11.8 (9.9, 13.5)	<0.001
BMI, kg/m^2^	18.3 (16.6, 20.6)	17.5 (15.5, 18.9)	19.9 (17.7, 21.4)	0.002
Clinical data				
Age at diagnosis, years, median (IQR)	4 (3, 6)	4 (3, 4)	5 (4, 6)	0.017
Corticosteroid treatment, *n* (%)	68 (93.2%)	38 (92.7%)	30 (93.8%)	0.503
Regime, *n* (%)				0.230
Daily deflazacort	4 (5.5%)	1 (2.4%)	3 (9.4%)	
Daily prednisone	61 (83.6%)	36 (87.8%)	25 (78.1%)	
Intermittent prednisone	3 (4.1%)	1 (2.4%)	2 (6.3%)	
Scoliosis, *n* (%)**				<0.001
None	29 (39.7%)	23 (56.1%)	6 (18.8%)	
Mild	39 (53.4%)	18 (43.9%)	21 (65.6%)	
Moderate	5 (6.8%)	0	5 (15.6%)	
Physical function				
Ambulation, *n* (%)				<0.001
Ambulant	60 (82.2%)	40 (97.6%)	20 (62.5%)	
Non-ambulant	13 (17.8%)	1 (2.4%)	12 (37.5%)	
Vignos grade, median (IQR)	2 (1, 3)	1 (1, 2)	3 (1, 8)	0.025
Brook grade, median (IQR)	1 (1, 1)	1 (1, 1)	1 (1, 3)	0.012
MFM scores (%), median (IQR)				
TS	80.2 (68.3, 88.0)	84.4 (77.6, 88.5)	69.8 (52.3, 87.0)	0.004
D1	53.8 (29.5,76.7)	61.5 (46.2, 78.2)	30.8 (5.75, 70.5)	0.002
D2	97.2 (94.4, 100.0)	97.2 (94.4, 100.0)	95.8 (79.2, 99.3)	0.017
D3	95.2 (92.9, 100.0)	95.2 (95.2, 100.0)	95.2 (90.5, 100.0)	0.191
Wheelchair use, *n* (%)				0.003
Never	54 (74.0%)	36 (87.8%)	18 (56.3%)	
Outdoor activities only	9 (12.3%)	4 (9.8%)	5 (15.6%)	
All daily activities	10 (13.7%)	1 (2.4%)	9 (28.1%)	
Orthotics use, *n* (%)				0.759
Never	39 (53.4%)	24 (58.5%)	15 (46.9%)	
Daytime	6 (8.2%)	3 (7.3%)	3 (9.4%)	
Nighttime	21 (28.8%)	10 (24.4%)	11 (34.4%)	
Daytime and nighttime	7 (9.6%)	4 (9.8%)	3 (9.4%)	
Fatigue				
PedsQL™ MFS, median (IQR)				
Overall fatigue score	62.5 (55.4, 67.6)	65.3 (61.2, 69.8)	55.6 (51.0, 62.5)	<0.001
General fatigue	66.7 (54.6, 79.6)	76.4 (64.1, 86.3)	56.3 (45.6, 66.7)	<0.001
Sleep/rest fatigue	58.3 (49.7, 66.8)	61.5 (54.2, 68.3)	54.4 (45.6, 64.0)	0.044
Cognitive fatigue	60.5 (50.9,70.1)	64.6 (55.6, 70.5)	56.3 (50.0, 69.4)	0.142
Psychological distress				
HADS, median (IQR)				
Total score	10 (8, 14)	9 (6.5, 10)	14 (10.3, 17.8)	<0.001
Anxiety score	6 (5, 8)	5 (4, 6)	8 (6, 10)	<0.001
Depression score	5 (3,6)	4 (2, 5)	6 (5, 8)	<0.001
HRQoL				
PedsQL™ 4.0 GCS, median (IQR)				
Total score	53.5 (43.8, 60.4)	57.3 (52.6, 68.5)	46.9 (42.3, 55.8)	<0.001
Physical health	46.4 (33.9, 60.9)	54.0 (46.4, 67.9)	39.3 (22.3, 49.1)	<0.001
Psychosocial health	57.2 (48.8, 67.0)	58.3 (55.0, 69.4)	53.9 (45.4, 60.8)	0.010
Emotional functioning	62.5 (50.0, 70.8)	63.0 (52.1, 73.0)	54.2 (45.9, 67.0)	0.046
Social functioning	60.0 (47.5, 75.0)	65.0 (55.0, 80.0)	57.5 (41.3, 65.0)	0.022
School functioning	50.0 (40.0, 60.0)	55.0 (45.0, 62.5)	50.0 (40.0, 55.0)	0.146

IQR, interquartile range; BMI, body mass index; MFM, motor function measure; TS, total score; D1, domain 1 (standing and transfers) of the motor function measure; D2, domain 2 (axial and proximal motor function) of the motor function measure; D3, domain 3 (distal motor function) of the motor function measure; PedsQL™ GSC, Pediatric Quality of Life Inventory™ Generic Core Scale; PedsQL™ MFS, Pediatric Quality of Life Inventory™ Multidimensional Fatigue Scale; HADS, Hospital Anxiety and Depression Scale.

*p-values calculated using Mann–Whitney test or chi-square test as appropriate.

**The severity of scoliosis is categorized as follows: mild (Cobb angle < 20°), moderate (Cobb angle between 20° and 40°), and severe (Cobb angle > 40°).

### 3.2 Predictors and pathways of pain outcomes at 1-year-follow-up

To identify baseline predictors of chronic pain at 1-year follow-up (*n* = 73), we first conducted a multivariable logistic regression analysis (Table 2). The model was statistically significant (*χ*^2^(5) = 44.30, *p* < 0.001), demonstrating strong explanatory power (Cox & Snell *R*^2^ = 0.534; Nagelkerke *R*^2^ = 0.716). After adjusting for baseline pain status, two variables emerged as significant independent predictors: physical function and psychological distress. Better physical function (MFM total score) was associated with lower odds of chronic pain (*B* = −0.073, *p* = 0.028; OR = 0.929, 95% CI [0.586, 0.988]), while higher psychological distress (HADS total score) was associated with increased risk (*B* = 0.407, *p* = 0.004; OR = 1.502, 95% CI [1.157, 5.508]). In contrast, neither fatigue (PedsQL™ MFS total score; *B* = −0.090, *p* = 0.162) nor HRQoL (PedsQL™ 4.0 total score; *B* = 0.003, *p* = 0.919) demonstrated a significant effect in the full model.

**Table 2:** Baseline predictors of pain outcomes identified via multivariable logistic regression.

Variables	*B*	S.E.	Wald	*p*	OR	95% CI
MFM total score	−0.073	0.033	4.838	0.028*	0.929	[0.586, 0.988]
HADS total score	0.407	0.142	8.216	0.004*	1.502	[1.157, 55.08]
PedsQL™ MFS total score	−0.090	0.064	1.957	0.162	0.914	[0.582, 1.077]
PedsQL™ 4.0 total score	0.003	0.034	0.010	0.919	1.003	[0.809, 1.146]
Baseline pain condition^**^	1.199	0.784	2.337	0.126	3.317	[0.317, 3,040]

*B*, unstandardized regression coefficient; S.E., standard error of the coefficient; Wald, Wald chi-square test statistic; OR, odds ratio; CI, confidence interval. MFM, motor function measure; PedsQL™, Pediatric Quality of Life Inventory™ Scale; PedsQL™ MFS, Pediatric Quality of Life Inventory™ Multidimensional Fatigue Scale; HADS, Hospital Anxiety and Depression Scale.

^*^p < 0.05 indicates statistical significance.

**Baseline pain condition is coded as 0 = no chronic pain, 1 = with chronic pain.

**Figure 2:**
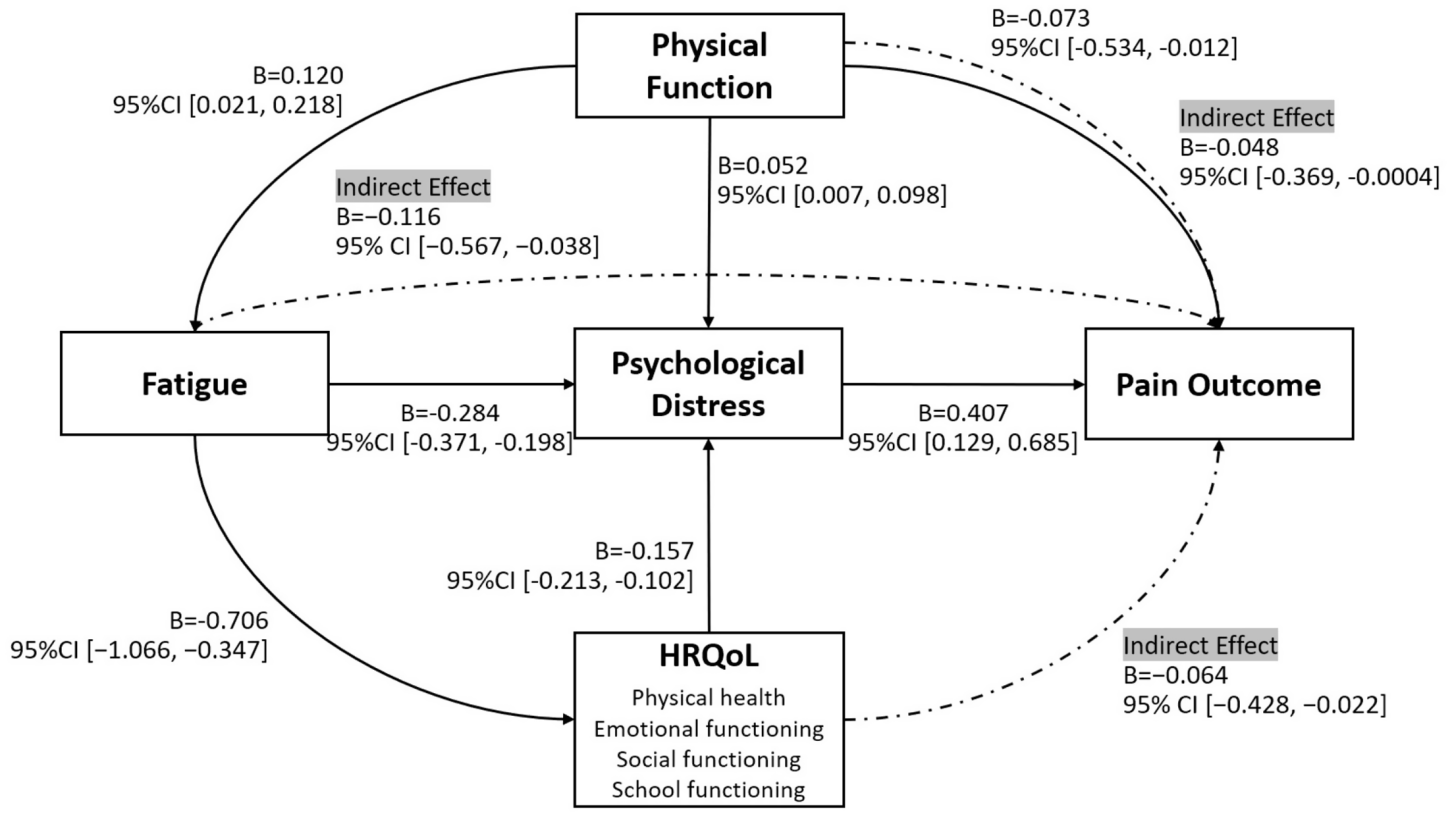
Integrated path model of baseline predictors influencing pain outcome at 1-year follow-up. Solid lines represent significant direct effects, while dashed lines indicate significant indirect effects. Coefficients (B) and 95% confidence intervals (CI) are shown for each pathway. HRQoL, health related quality of life.

To examine potential mediation effects, we conducted a series of analyses using PROCESS Model 4, adjusting for relevant baseline covariates. Fatigue significantly predicted higher psychological distress (*B* = −0.284, *p* < 0.001, 95% CI [−0.371, −0.198]), which in turn was associated with an increased risk of chronic pain (*B* = 0.407, *p* = 0.004, 95% CI [0.129, 0.685]). The indirect effect of fatigue on pain outcome via psychological distress was significant (indirect effect = −0.116, 95% CI [−0.567, −0.038]). HRQoL also demonstrated a significant indirect effect through psychological distress. Specifically, the total HRQoL score yielded an indirect effect of −0.064 (95% CI [−0.428, −0.022]), with similar effects observed across subdomains: physical health (−0.031, 95% CI [−0.198, −0.008]), emotional functioning (−0.059, 95% CI [−0.314, −0.025]), social functioning (−0.028, 95% CI [−0.171, −0.009]), and school functioning (−0.033, 95% CI [−0.136, −0.012]). In addition, physical function showed a modest indirect effect through psychological distress (indirect effect = −0.048, 95% CI [−0.369, −0.0004]). Figure 2 presents an integrated path model summarizing both direct and indirect pathways linking baseline predictors to chronic pain outcomes at 1-year follow-up.

### 3.3 Predictors of pain burden at 1-year follow-up

To clarify factors contributing to chronic pain burden, we assessed baseline predictors of pain severity (average NPRS score) and pain interference (PIS-BPI total score) at the 1-year follow-up. Spearman’s correlation analyses (*n* = 32) showed that pain severity was positively correlated with age (*ρ* = 0.50, *p* < 0.001), BMI (*ρ* = 0.38, *p* < 0.001), HRQoL (*ρ* = 0.56, *p* < 0.001), psychological distress (*ρ* = 0.68, *p* < 0.001), and fatigue (*ρ* = 0.53, *p* < 0.001), and negatively correlated with physical function (*ρ* = −0.36, *p* = 0.002). Pain interference showed a similar pattern, with positive correlations with age (*ρ* = 0.43, *p* < 0.001), BMI (*ρ* = 0.34, *p* = 0.004), HRQoL (*ρ* = 0.52, *p* < 0.001), psychological distress (*ρ* = 0.68, *p* < 0.001), and fatigue (*ρ* = 0.57, *p* < 0.001), and a negative correlation with physical function (*ρ* = −0.26, *p* = 0.027).

Multivariable linear regression identified psychological distress and fatigue as robust independent predictors of pain burden. Psychological distress was associated with higher pain severity (*B* = 0.208, *p* < 0.001; 95% CI [0.105, 0.307]) and pain interference (*B* = 0.193, *p* < 0.001; 95% CI [0.100, 0.279]). Similarly, fatigue was associated with greater pain severity (*B* = −0.054, *p* = 0.037; 95% CI [−0.107, −0.006]) and pain interference (*B* = −0.047, *p* = 0.020; 95% CI [−0.085, −0.008]). Physical function predicted pain severity (*B* = −0.036, *p* = 0.001; 95% CI [−0.057, −0.012]) but not pain interference, while HRQoL showed no significant associations in either model (Table 3).

**Table 3:** Baseline predictors of pain burdens identified via multivariable linear regression.

Variables	*B*	S.E.	*β*	*t*	*p*	95% CI
Pain severity						
MFM total score	−0.036	0.010	−0.284	−3.444	0.001*	[−0.057, −0.012]
HADS total score	0.208	0.054	0.426	3.836	0.000*	[0.105, 0.307]
PedsQL™ MFS total score	−0.054	0.025	−0.212	−2.127	0.037*	[−0.107, 0.006]
PedsQL™ 4.0 total score	−0.029	0.015	−0.184	−1.889	0.063	[−0.063, 0.000]
Pain interference						
MFM total score	−0.013	0.008	−0.132	−1.561	0.123	[−0.032, 0.008]
HADS total score	0.193	0.043	0.515	4.512	0.000*	[0.100, 0.279]
PedsQL™ MFS total score	−0.047	0.020	−0.243	−2.374	0.020*	[−0.085, −0.008]
PedsQL™ 4.0 total score	−0.016	0.012	−0.128	−1.283	0.204	[−0.040, 0.007]

*B*, unstandardized coefficient; S.E., standard error of the coefficient; *β*, standardized coefficients; CI, confidence interval. MFM, motor function measure; PedsQL™, Pediatric Quality of Life Inventory™ Scale; PedsQL™ MFS, Pediatric Quality of Life Inventory™ Multidimensional Fatigue Scale; HADS, Hospital Anxiety and Depression Scale.

*p < 0.05 indicates statistical significance.

## 4 Discussion

This prospective study provides a 1-year follow-up of pain in DMD, moving beyond cross-sectional estimates to describe how chronic pain develops and changes over time. The path model indicates that chronic pain in DMD reflects a biopsychosocial process. Baseline physical function and psychological distress independently predicted pain onset and persistence, with psychological distress mediating the effects of fatigue and reduced HRQoL. These findings support the biopsychosocial model in this population and delineate pathways linking physical, emotional, and functional factors to pain trajectories (19).

Integrating the present longitudinal findings with our recent cross-sectional investigation of somatosensory profiles in DMD yields a more nuanced, two-tiered framework for understanding chronic pain development. Our prior QST study demonstrated that individuals with DMD, regardless of pain status, exhibit generalized hyperalgesia, manifested as decreased pressure pain thresholds, compared to healthy controls (9). This indicates that heightened sensory sensitivity, potentially reflecting central sensitization (20), may be an intrinsic pathophysiological characteristic of the disease. However, the inability of QST measures to consistently distinguish DMD individuals with and without chronic pain raises an important question: if sensory profiles are largely similar, what drives the development of a clinical pain syndrome? The current findings suggest that psychosocial factors may play a key role. While a sensitized nervous system may create a vulnerable background for pain, psychological distress, fatigue, and related psychosocial factors likely act as catalysts, shaping this vulnerability into the subjective experience and clinical burden of chronic pain (21).

A key finding of this study was a direct association between impaired physical function and higher risk of chronic pain. This aligns with previous research suggesting that progressive muscle weakness in DMD may contribute to musculoskeletal pain via biomechanical stress and joint deformities (6–8, 22). Furthermore, our mediation analysis suggests that this relationship is partially influenced by psychological distress. This indicates that physical decline in DMD may act not only as a biological stressor but also as an emotional one, with the resulting distress potentially amplifying pain perception (23). Therefore, interventions focusing solely on physical symptoms may have limited effectiveness if the accompanying psychological burden is not also addressed.

Psychological distress emerged as a central predictor of chronic pain risk, consistent with prior research on the role of affective symptoms in the development and persistence of chronic pain (21, 23, 24). In individuals with DMD, such distress naturally arises from living with a progressive, physically disabling, and ultimately life-limiting condition (25). The resulting psychosocial burden is substantial, cumulative, and exerts a sustained impact on overall wellbeing (26). Previous work in this cohort identified clinically significant anxiety (24%) and depressive (11%) symptoms, highlighting their elevated psychological vulnerability (5, 9). Mediation analyses in the current study further clarify the multifaceted role of psychological distress, showing that it not only directly influences chronic pain risk but also mediates the effects of physical function, fatigue, and HRQoL on pain outcomes. Given the cross-sectional nature of baseline assessments, this mediation findings are exploratory and should not be interpreted as evidence of causal pathways. Together, these findings reinforce the biopsychosocial model, emphasizing psychological factors as key intermediaries in shaping the pain experience in chronic neuromuscular conditions such as DMD.

Fatigue and HRQoL played important indirect roles in chronic pain. Neither factor directly predicted pain onset, but both influenced outcomes through psychological distress. The lack of a direct pathway from fatigue to pain suggests that fatigue may not be a primary nociceptive driver in DMD (2); instead, its burden appears to increase pain vulnerability via emotional distress. Similarly, the impact of reduced HRQoL, especially in social and school domains, was fully mediated by psychological distress, indicating that challenges such as social isolation or academic difficulties do not directly cause pain, but contribute to a state of emotional vulnerability that heightens pain risk. In this context, fatigue and poor HRQoL act as upstream contributors, whose effects on pain are channeled through their influence on psychological wellbeing.

In addition to predicting pain onset, this study identified key factors influencing long-term pain burden. Psychological distress and fatigue were strong predictors of both pain severity and interference, highlighting the significant role of affective and somatic-energetic factors. Notably, poorer physical function was associated with greater pain severity, likely reflecting its direct link to nociceptive sources (7), but did not independently predict pain interference. This suggests that, once pain is established, its impact on daily functioning may be more strongly shaped by psychological state and energy levels than by baseline physical limitations. Overall, these findings depict a multidimensional pain experience in DMD, with distinct pathways contributing to sensory versus functional aspects of pain.

### 4.1 Clinical implications

Our findings support a shift in the clinical management of pain in DMD, from reactive treatment to a proactive, mechanism-informed approach focused on risk stratification. While biological vulnerability to pain may be inherent in DMD, psychosocial factors appear to be key determinants in the development of clinical pain. Despite strong evidence and formal guidelines advocating for psychosocial management, a substantial care gap remains: although 83% of patients report mental health concerns, only 24.3% access psychosocial services, and half of those with identified needs receive no referrals or interventions (27). This ongoing failure to address psychological distress leaves a key pathogenic factor unmitigated, potentially contributing to the high prevalence of chronic pain in DMD.

To enhance translational impact, we recommend a preventive, biopsychosocial approach to pain management in DMD. Routine screening for psychological distress and fatigue should be conducted at diagnosis and during regular follow-up visits (e.g., every 6–12 months) using brief, validated tools such as the HADS or age-adapted alternatives. Children who screen positive should be promptly referred to pediatric psychologists or multidisciplinary teams for assessment and evidence-based interventions, including cognitive-behavioral therapy (28, 29). This approach aligns with international guidelines advocating a biopsychosocial model for pediatric chronic pain, emphasizing early identification, timely intervention, and integration of psychosocial care within routine multidisciplinary management to prevent chronic pain and improve overall wellbeing.

Furthermore, addressing fatigue and supporting social and academic functioning should be viewed as essential upstream interventions that strengthen emotional resilience and indirectly reduce pain risk, rather than as isolated objectives. While maintaining physical function remains crucial for managing nociceptive pain and preserving quality of life, integrating psychosocial care addresses a critical unmet need. A comprehensive, biopsychosocial approach is therefore necessary for truly effective clinical management of pain in DMD.

### 4.2 Study limitations

Several limitations of this study should be acknowledged. First, the modest sample size (*n* = 73) limited statistical power and the generalizability of our findings. This contributed to wide odds ratios and confidence intervals for some predictors (e.g., baseline pain condition in Table 2), reflecting low event counts in certain subgroups and potentially reducing estimate stability. Model diagnostics were carefully evaluated and key covariates were controlled to minimize overfitting; nonetheless, future studies with larger samples could benefit from simplified models or penalized regression approaches to enhance robustness. Second, some assessment tools have limitations in this population. Although the HADS has demonstrated acceptable internal consistency and construct validity in children and adolescents from approximately 8 years of age, it has not been formally validated specifically in children with DMD aged 8–10 years. Accordingly, scores in this subgroup should be interpreted cautiously and in conjunction with clinical assessment. Third, pain trajectories were dichotomized due to small sample sizes in the new-onset (*n* = 7) and recovered (*n* = 9) groups, which limited power for multigroup comparisons. Baseline pain status was included as a covariate in the logistic regression models to preserve longitudinal information, partially accounting for differences between participants with and without baseline pain. However, this approach may obscure heterogeneity between new-onset and persistent pain groups; the full distribution of pain trajectories is retained and illustrated using violin plots in Figure 1. Finally, the baseline assessment was cross-sectional, and reliance on self-reported data introduces potential recall and response biases, although validated instruments and caregiver corroboration likely mitigated this concern. Consequently, the mediation findings should be considered exploratory rather than confirmatory, as causal inferences cannot be drawn and the temporal relationship between psychological distress and fatigue remains uncertain. Future studies with multiple assessment waves, such as cross-lagged panel models, are needed to examine directional and potentially reciprocal associations more rigorously.

## 5 Conclusion

This prospective study identifies psychological distress and impaired physical function as key, independent contributors to chronic pain outcomes in DMD, with psychological distress further mediating the effects of fatigue and HRQoL. Physical function, psychological distress, and fatigue also predicted broader pain burden. These findings support a biopsychosocial model in which psychosocial factors play a central role in translating underlying biological vulnerability, such as sensory hypersensitivity, into a clinical pain syndrome. The results underscore the need for a shift toward proactive, multidomain risk stratification and early, personalized interventions to prevent chronic pain and enhance quality of life in this population.

## Data Availability

The raw data supporting the conclusions of this article will be made available by the authors, without undue reservation.
